# Investigating ethylene oxide exposure and its associations with kidney indicators and lipid profiles: the mediating effect of HDL in NHANES 2013-2020

**DOI:** 10.3389/fphys.2025.1523940

**Published:** 2025-05-22

**Authors:** Yiheng Luo, Duo Xu, Weiyong Zhang, Kai Wang, Mingqin Kuang, Yueyang Liu

**Affiliations:** ^1^ The First School of Clinical Medicine, Southern Medical University, Guangzhou, China; ^2^ School of Public Health, Southern Medical University, Guangzhou, China; ^3^ Department of Internal Medicine, Shenzhen TCM Anorectal Hospital (Futian), Shenzhen, China; ^4^ Department of Gynecology and Oncology, Ganzhou Cancer Hospital, Ganzhou, China; ^5^ Department of Gynecology, Guangdong Provincial People’s Hospital (Guangdong Academy of Medical Sciences), Southern Medical University, Guangzhou, China

**Keywords:** ethylene oxide, kidney parameters, lipid profiles, epidemiology, national health and nutrition examination survey

## Abstract

**Introduction:**

Ethylene oxide (EO) exposure has been associated with various health conditions, such as cancer and cardiovascular diseases. However, its potential relationships with kidney function and lipid profiles require further investigation.

**Methods:**

This study analyzed data from 3,500 US adults participating in NHANES 2013–2020. EO exposure was assessed using hemoglobin adducts of EO (HbEO) as a biomarker. Associations with kidney and lipid parameters were evaluated using multivariate linear regression models. Mediation analysis was performed to explore the role of high-density lipoprotein (HDL) in these associations.

**Results:**

Higher HbEO levels were significantly associated with decreased albumin (Alb) (β = −0.79, 95% CI: −1.15, −0.43) and increased blood urea nitrogen in the second and third quartiles (Q2: (β = 0.79, 95% CI: 0.34, 1.24; Q3: (β = 0.81, 95% CI: 0.35, 1.27). Uric acid (UA) showed an inverse association with the highest quartile of HbEO (β = −0.23, 95% CI: −0.36, −0.09). Log10-transformed HbEO levels were negatively associated with Alb, UA, and the UA/serum creatinine ratio. Regarding lipids, no significant associations were found with triglycerides, total cholesterol, or LDL. However, EO exposure was negatively associated with HDL levels (β = −3.57, 95% CI: −5.18, −1.96). Mediation analysis revealed that HDL mediated 6.51% of the association between EO and Alb, 12.44% with UA (inverse), and 11.01% with urinary creatinine.

**Discussion:**

EO exposure is significantly associated with alterations in kidney function and HDL levels. HDL's mediating role suggests a potential mechanism linking EO to renal biomarkers, warranting further mechanistic investigation.

## Introduction

Ethylene oxide (EO), a highly toxic industrial compound, is widely used as a chemical precursor and in sterilization, fumigation, cosmetics, and tobacco processing (Chemical agents and related occupations, 2012; Jain, 2020). Animal studies have demonstrated that high concentrations of EO can significantly increase tumor incidence (Kirman et al., 2021). Epidemiological investigations have further suggested that prolonged exposure to EO may contribute to the development of chronic diseases such as diabetes, hypertension, cardiovascular disorders, neurological impairments, and various cancers (Guo et al., 2021; Estrin et al., 1987; Steenland et al., 2004; Sun et al., 2025). However, findings from large-scale human studies remain inconsistent, with some research reporting no clear association between EO exposure and increased cancer risk (Jain, 2020). These findings highlight the need for more comprehensive studies on EO’s health impacts.

Moreover, individuals are exposed to EO through various pathways, including endogenous production via bacterial activity in the gastrointestinal tract or systemic synthesis in the liver (Swenberg et al., 2011); metabolism of exogenous ethylene sources, including vehicle exhaust, fires, and cigarette smoke (Filser et al., 2013); and inhalation of high EO concentrations near industrial or sterilization facilities (Olaguer et al., 2019). Given these findings, it is essential to understand the effects of EO on the human body and to devise effective public health interventions. Due to EO’s multifaceted exposure pathways, a robust biomarker is required to assess cumulative internal exposure. Hemoglobin adducts of ethylene oxide (HbEO) serve as a biomarker for assessing long-term EO exposure in humans (Zhou C. et al., 2024).

Several studies have reported associations between ethylene oxide (EO) exposure and adverse renal outcomes, with elevated HbEO levels positively correlating with an increased risk of kidney stones, chronic kidney disease, and proteinuria (Song et al., 2023; Wu et al., 2024; Zhou W. et al., 2024). However, the association between EO and kidney function remains unclear. Given that blood urea nitrogen (BUN), uric acid (UA), serum creatinine (Scr), serum albumin (Alb), urinary creatinine (Ucr), and urinary albumin (Ualb) are well-validated biomarkers of kidney function, these parameters were systematically analyzed in the present study (Sanders et al., 2019; Zhang et al., 2023; Ji et al., 2022). Additionally, the ratio of UA to Scr (UA/Scr) has emerged as a novel biomarker that provides a more accurate reflection of endogenous UA levels and assists in the evaluation of renal function (Gu et al., 2017).

The impact of EO on lipid metabolism is equally significant, as dysregulated lipid levels play a crucial role in the onset of cardiovascular diseases (CVDs) and various other health conditions (White-Al Habeeb et al., 2023). Zeng et al. (Zeng et al., 2021) established a positive link between EO exposure and an increased prevalence of CVDs, which are strongly linked to serum total cholesterol (TC), triglycerides (TG), and low-density lipoprotein (LDL). High-density lipoprotein (HDL), known for delaying atherosclerosis progression, cannot induce regression (Zhu et al., 2022). Additionally, the atherogenic index of plasma (AIP), a novel and more robust indicator of lipid imbalances, has emerged as a key predictor of CVD risk, especially in populations with metabolic abnormalities (Min et al., 2024).

HDL levels are closely linked to kidney disease (Vaziri, 2016) and may mediate the relationship between lipid parameters and renal function (Sun et al., 2015). While the role of HDL in kidney disease has been extensively characterized, its potential mediating role in the association between EO exposure and changes in kidney function remains unclear. Investigating this mediation may offer novel mechanistic insights into how EO affects physiological processes, particularly cardiovascular and renal health.

The underlying pathophysiology of EO-mediated organ toxicity has been primarily characterized in animal models, while their human implications require further investigation. This study systematically evaluated the potential nephrotoxic and dyslipidemic effects of EO exposure in human populations, hypothesizing that higher EO exposure is associated with impaired renal markers, and that this relationship is partially mediated by changes in lipid metabolism, particularly HDL. Using data from the 2013–2020 National Health and Nutrition Examination Survey (NHANES), our study investigated the relationship between human EO levels and kidney function while assessing the potential mediating role of HDL.

## Methods

### Study population

This study leveraged data from the NHANES conducted between 2013 and 2020, as HbEO measurements were only available during this period. Initially, 35,706 participants were enrolled. To refine the population, we excluded 27,209 individuals with missing HbEO data, resulting in 8,497 participants. To minimize potential bias and ensure the study population represented adults with stable metabolic profiles, individuals under the age of 20 were excluded, resulting in the removal of an additional 2,396 participants, leaving 6,101 eligible subjects.

**FIGURE 1:**
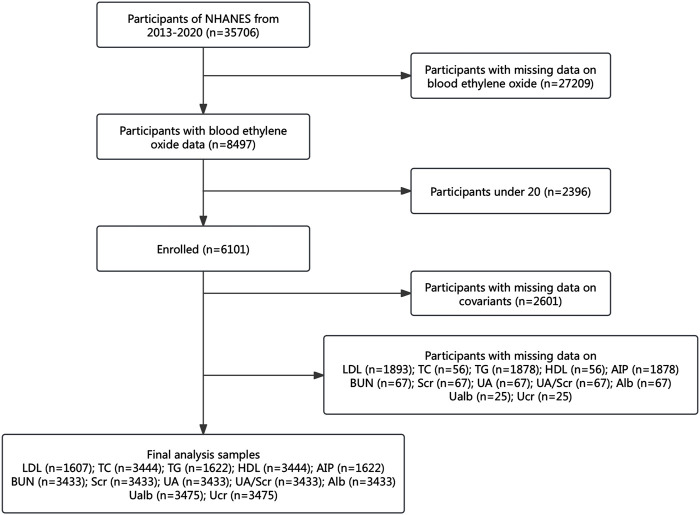
Study population flowchart. NHANES: National Health and Nutrition Examination Survey; Alb: serum albumin; BUN: blood urea nitrogen; UA: uric acid; Scr: serum creatinine; Ucr: urinary creatinine; Ualb: urinary albumin; UA/Scr ratio: serum uric acid divided by serum creatinine; HDL: high-density lipoprotein; TC: total cholesterol; TG: triglycerides; LDL: low-density lipoprotein; AIP: atherogenic index of plasma.

Further exclusions were made for participants with incomplete covariate data, including gender, age, race, Poverty-Income Ratio (PIR), education level, alcohol consumption, smoking status, Body Mass Index (BMI), vigorous work activity, diabetes, and hypertension. This step eliminated 2,601 subjects, resulting in a final analysis group of 3,500 individuals. Participants without available data on kidney parameters and lipid profiles were also excluded, with specific details outlined in Figure 1. The Ethics Review Board of the National Center for Health Statistics approved the NHANES study protocol, and all participants provided their written informed consent (http://www.cdc.gov/nchs/nhanes).

### Assessment of ethylene oxide

The assessment of EO exposure was conducted by measuring hemoglobin adducts, specifically N-terminal valine adducts, using a modified Edman reaction for enhanced sensitivity. Quantification was performed via high-performance liquid chromatography coupled with tandem mass spectrometry, allowing for accurate detection of EO from both exogenous and endogenous sources. More information is available on the NHANES website (https://wwwn.cdc.gov/nchs/data/nhanes/2017-2018/labmethods/ETHOX-J-MET-508.pdf).

### Kidney parameters and lipid profiles

We assessed kidney parameters, including Alb (g/L), BUN (mg/dL), UA (mg/dL), Scr (mg/dL), Ucr (mg/dL), Ualb (mg/L), and the UA/Scr ratio, calculated as serum uric acid (mg/dL) divided by serum creatinine (mg/dL). Lipid parameters included HDL (mg/dL), TC (mg/dL), TG (mg/dL), LDL (mg/dL), and AIP, calculated as log10 (TG/HDL).

### Covariates

To reduce confounding bias and ensure the consistency and comparability of our results, we adjusted for several covariates based on prior studies, including gender, age, race, PIR, education level, alcohol consumption, smoking status, BMI, vigorous work activity, diabetes, and hypertension (Zhou C. et al., 2024; Politis et al., 2021).

Participants were classified into five racial categories: Mexican American, Other Hispanic, Non-Hispanic White, Non-Hispanic Black, and Other Race, which includes multi-racial individuals. Education level was classified into three categories: below high school, high school, and above high school (Zhu et al., 2022). For alcohol consumption, data were obtained from the questionnaire asking the average number of alcoholic drinks per day in the past 12 months. Smoking status was classified according to whether participants had smoked at least 100 cigarettes in their lifetime (Zhu et al., 2022). BMI was categorized into three groups: <25 kg/m^2^ (normal weight), 25–29.9 kg/m^2^ (overweight), and ≥30 kg/m^2^ (obese) for all participants (Li et al., 2024).

Data on vigorous work activity were gathered through questionnaires, inquiring whether participants’ jobs involved high-intensity physical activity, such as carrying heavy loads or construction work, that significantly increased breathing or heart rate for at least ten continuous minutes. For diabetes and hypertension, we did not rely solely on direct measurements of blood glucose and blood pressure, as single measurements are insufficient for diagnosing these two conditions. Instead, we included self-reported data, asking whether participants had ever received a physician diagnosis of diabetes or hypertension.

### Statistical analysis

We applied a log10 transformation to HbEO to account for its skewed distribution and then categorized the log10-transformed values into quartiles. Only participants with complete data on all variables used in the models were included in the analysis, and a complete-case analysis approach was adopted. To investigate the independent associations between HbEO and kidney parameters as well as lipid profiles, multivariate logistic regression models were employed, incorporating both continuous log10-transformed HbEO and quartile categories across three different models: Model 1 with no adjustments, Model 2 adjusted for gender, race, age, and BMI, and Model 3 with full adjustments for additional covariates. Multicollinearity among independent variables was assessed using variance inflation factors, and no significant multicollinearity was detected (ranged from 1.1 to 1.5). Smooth curve fitting and restricted cubic spline (RCS) regression were utilized to evaluate further the nonlinear relationships involving HbEO and the various parameters, with four knots placed at the 5th, 35th, 65th, and 95th percentiles of HbEO distribution. Additionally, we conducted mediation analyses to investigate whether lipid profiles mediate the effect of HbEO on kidney parameters, aiming to determine the role of lipid levels in the relationship between HbEO and kidney function. Multiple linear regression and mediation analyses were performed using Empower version 4.1 (www.empowerstats.com) and R version 4.2.0. Restricted cubic spline modeling and curve plotting were conducted with R version 4.4.0 and Zstats 1.0 (www.zstats.net).

## Results

### Baseline characteristics

Our analytic sample included 3,500 participants. The median age was 47.0 years, and 52.1% of participants were male. The majority were Non-Hispanic White (40.8%) and completed education beyond high school (61.6%). The median HbEO level was 22.3 pmol/g Hb. Participants were categorized by BMI as 27.1% normal weight, 31.8% overweight, and 41.0% obese. Nearly half of the participants were smokers (47.4%), with a median alcohol consumption of 2.0 drinks per week. Additionally, 34.3% had hypertension, and 14.7% had diabetes. Further details are provided in Table 1.

**TABLE 1:** Survey-weighted, sociodemographic and health status characteristics of adult NHANES 2013–2020 participants with available blood ethylene oxide data (n = 3500).

Variable	Median (IQR) or N (%)
Age, years	47.0 (33.0, 61.0)
Gender	
Male, %	1825 (52.1%)
Female, %	1675 (47.8%)
Education level, %	
Below high school	504 (14.4%)
High school	837 (23.9%)
Above high school	2159 (61.6%)
Race/ethnicity, %	
Mexican American	461 (13.1%)
Other Hispanic	339 (9.6%)
Non-Hispanic White	1431 (40.8%)
Non-Hispanic Black	788 (22.5%)
Other race	481 (13.7%)
Poverty, %	594 (17.0%)
Smoking status, %	1661 (47.4%)
Alcohol consumption, %	2.0 (1.0, 3.0)
Body mass index, kg/m^2^	
Normal weight	949 (27.1%)
Overweight	1113 (31.8%)
Obese	1438 (41.0%)
Physical activity, %	852 (24.3%)
Hypertension, %	1203 (34.3%)
Diabetes, %	516 (14.7%)
HbEO, pmol/g Hb	22.3 (15.9, 56.7)
Serum albumin, mg/dL	42.0 (40.0, 45.0)
Urinary albumin, mg/dL	7.8 (4.0, 16.0)
Urinary creatinine, mg/dL	108.0 (60.0, 166.0)
Blood urea nitrogen, mg/dL	13.0 (11.0, 17.0)
Uric acid, mg/dL	5.4 (4.4, 6.3)
Creatinine, refrigerated serum, mg/dL	0.9 (0.7, 1.0)
Serum uric acid/serum creatinine ratio	6.2 (5.2, 7.3)
Triglyceride, mg/dL	91.0 (60.0, 136.0)
Total cholesterol, mg/dL	185.0 (159.0, 213.0)
Low-density lipoprotein cholesterol, mg/dL	107.0 (85.0, 132.0)
High-density lipoprotein cholesterol, mg/dL	52.0 (42.0, 63.0)
Plasma atherogenic index, AIP	0.2 (0, 0.5)

Data are presented as median (IQR) or N (%); sampling weights were applied for calculation of demographic descriptive statistics. N reflects the study sample, whereas percentages reflect the survey-weighted data.

### Association between HbEO and kidney parameters

Table 2 illustrates the association between HbEO and kidney parameters. In this analysis, log10-transformed HbEO was significantly associated with lower Alb levels. In the fully adjusted model, the highest quartile of HbEO exposure showed a β = −0.79 (95% CI: −1.15, −0.43), p < 0.0001. Continuous log10-transformed HbEO also demonstrated a significant inverse association (β = −0.72, 95% CI: −1.00, −0.44, p < 0.0001), further underscoring the consistent relationship between HbEO exposure and reduced Alb levels. For BUN, higher HbEO exposure in Q2 and Q3 was positively associated with increased levels. In Q2, the fully adjusted model indicated a significant positive relationship with blood urea nitrogen (BUN) levels, showing a β of 0.79 (95% CI: 0.34, 1.24, p = 0.0006). The association persisted in Q3, with a β of 0.81 (95% CI: 0.35, 1.27, p = 0.0006). However, continuous log10-transformed HbEO exposure revealed a significant inverse association with BUN, demonstrating a β of −0.52 (95% CI: −0.94, −0.10, p = 0.0159), indicating a complex relationship between HbEO exposure and BUN levels. Uric acid (UA) levels also exhibited a significant inverse association with HbEO exposure. In the fully adjusted model, the highest quartile (Q4) showed a β of −0.23 (95% CI: −0.36, −0.09, p = 0.0009). Additionally, continuous log10-transformed HbEO demonstrated a significant inverse correlation with UA levels (β = −0.27, 95% CI: −0.38, −0.17, p < 0.0001). The UA/Scr ratio displayed a similar trend, with Q4 exhibiting a β of −0.32 (95% CI: −0.50, −0.14, p = 0.0005), and continuous HbEO also showed a significant relationship (β = −0.35, 95% CI: −0.49, −0.21, p < 0.0001).

**TABLE 2:** Multiple linear regression associations of HbEO with kidney parameters in adults.

Kidney parameters	Model	Continuous log10-transformed EO	Quartile1	Quartile2	Quartile3	Quartile4	p for trend
			β	β(95%CI)	β(95%CI)	β(95%CI)	
Ucr(n = 3475)	Model 1	16.68 (10.97, 22.38)***	0.00(Ref.)	2.39 (−5.12, 9.90)	7.05 (−0.46, 14.56)	21.52 (14.00, 29.03)***	0.0541
	Model 2	7.66 (2.16, 13.17)**	0.00(Ref.)	1.29 (−5.71, 8.30)	4.95 (−2.14, 12.03)	9.32 (2.11, 16.54)*	
	Model 3	6.43 (−0.11, 12.98)	0.00(Ref.)	1.45 (−5.60, 8.50)	4.96 (−2.23, 12.15)	7.91 (−0.39, 16.22)	
BUN(n = 3433)	Model 1	−1.27 (−1.65, −0.89)***	0.00(Ref.)	0.84 (0.33, 1.34)***	0.56 (0.06, 1.07)*	−1.11 (−1.62, −0.61)***	0.0159
	Model 2	−0.79 (−1.15, −0.43)***	0.00(Ref.)	0.83 (0.38, 1.29)***	0.87 (0.41, 1.33)***	−0.52 (−0.98, −0.05)*	
	Model 3	−0.52 (−0.94, −0.10)*	0.00(Ref.)	0.79 (0.34, 1.24)***	0.81 (0.35, 1.27)***	−0.26 (−0.79, 0.28)	
UA (n = 3433)	Model 1	−0.12 (−0.22, −0.01)*	0.00(Ref.)	0.10 (−0.04, 0.23)	0.09 (−0.05, 0.22)	−0.02 (−0.16, 0.11)	<0.0001
	Model 2	−0.16 (−0.26, −0.07)***	0.00(Ref.)	0.03 (−0.09, 0.14)	0.06 (−0.06, 0.17)	−0.12 (−0.24, 0.00)	
	Model 3	−0.27 (−0.38, −0.17)***	0.00(Ref.)	0.05 (−0.06, 0.17)	0.06 (−0.05, 0.18)	−0.23 (−0.36, −0.09)***	
Scr(n = 3433)	Model 1	0.04 (0.01, 0.06)***	0.00(Ref.)	0.02 (−0.01, 0.05)	0.02 (−0.01, 0.05)	0.05 (0.02, 0.08)***	0.2342
	Model 2	0.00 (−0.02, 0.02)	0.00(Ref.)	0.01 (−0.01, 0.04)	0.02 (−0.01, 0.04)	0.01 (−0.02, 0.04)	
	Model 3	0.01 (−0.01, 0.04)	0.00(Ref.)	0.01 (−0.01, 0.04)	0.02 (−0.01, 0.04)	0.02 (−0.01, 0.05)	
UA/Scr(n = 1622)	Model 1	−0.36 (−0.48, −0.24)***	0.00(Ref.)	0.02 (−0.14, 0.18)	0.03 (−0.13, 0.19)	−0.31 (−0.47, −0.15)***	<0.0001
	Model 2	−0.17 (−0.29, −0.05)**	0.00(Ref.)	−0.03 (−0.18, 0.12)	−0.01 (−0.16, 0.15)	−0.13 (−0.29, 0.02)	
	Model 3	−0.35 (−0.49, −0.21)***	0.00(Ref.)	−0.01 (−0.16, 0.14)	−0.01 (−0.16, 0.15)	−0.32 (−0.50, −0.14)***	
Alb(n = 3433)	Model 1	−0.42 (−0.67, −0.17)**	0.00(Ref.)	−0.43 (−0.76, −0.09)*	−0.30 (−0.63, 0.04)	−0.53 (−0.86, −0.19)**	<0.0001
	Model 2	−0.81 (−1.05, −0.57)***	0.00(Ref.)	−0.46 (−0.76, −0.16)**	−0.36 (−0.66, −0.05)*	−0.97 (−1.28, −0.66)***	
	Model 3	−0.72 (−1.00, −0.44)***	0.00(Ref.)	−0.46 (−0.76, −0.16)**	−0.34 (−0.65, −0.03)*	−0.79 (−1.15, −0.43)***	
Ualb (n = 3475)	Model 1	6.87 (−13.99, 27.74)	0.00(Ref.)	4.27 (−23.21, 31.76)	32.89 (5.43, 60.36)*	7.33 (−20.18, 34.83)	0.8118
	Model 2	10.44 (−11.24, 32.12)	0.00(Ref.)	1.07 (−26.49, 28.64)	32.60 (4.73, 60.46)*	9.53 (−18.84, 37.89)	
	Model 3	3.10 (−22.39, 28.59)	0.00(Ref.)	−0.98 (−28.43, 26.47)	22.95 (−5.03, 50.93)	−3.34 (−35.65, 28.97)	

Model 1 was not adjusted.

Model 2 was adjusted for age, gender, race, and BMI.

Model 3 was adjusted as Model 2 plus education level, PIR, vigorous work activity, smoking status, alcohol consumption, diabetes, and hypertension.

Ucr: Urinary creatinine; BUN: blood urea nitrogen; UA: uric acid; Scr: serum creatinine; UA/Scr: UA/Scr ratio; Alb: serum albumin; Ualb: urinary albumin; Ref: reference; *p < 0.05, **p < 0.01, and ***p < 0.001.

Although the association with Ucr was marginally significant, a potential upward trend was observed (β = 7.91, 95% CI: −0.39, 16.22, p = 0.0619) in the highest exposure quartile. Continuous log10 HbEO also revealed a significant positive relationship with Ucr (β = 6.43, 95% CI: −0.11, 12.98, p = 0.05). However, no significant association was observed for Scr, with a β = 0.02 (95% CI: −0.01, 0.05), p = 0.1623.

**FIGURE 2:**
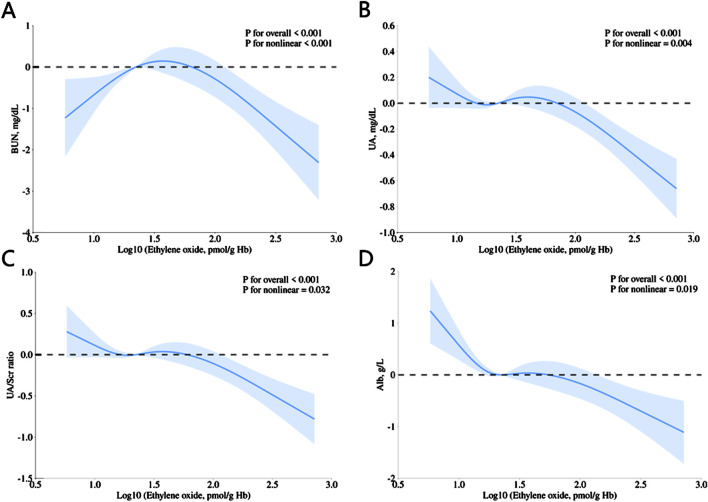
Restricted cubic spline (RCS) plots showing the association of HbEO levels with **(A)** BUN, **(B)** UA, **(C)** UA/Scr ratio, and **(D)** Alb. All models were adjusted for age, gender, education level, race, PIR, vigorous work activity, BMI, smoking status, alcohol consumption, diabetes, and hypertension.

In addition, the cubic spline analysis (with log10-transformed HbEO as a continuous variable) demonstrated significant nonlinear associations between HbEO and multiple kidney parameters, including BUN (p for nonlinear <0.001), UA (p for nonlinear = 0.004), UA/Scr (p for nonlinear = 0.032), and Alb (p for nonlinear = 0.019), as illustrated in Figure 2. These findings highlight that the relationship between HbEO and these parameters is nonlinear, indicating a complex dose-response trend rather than a straightforward linear pattern.

### Association between HbEO and lipid profiles

In the regression analysis shown in Table 3, a significant inverse relationship between HDL and HbEO exposure was observed across both log-transformed continuous HbEO and quartiles. In the unadjusted model, participants in the highest quartile (Q4) of HbEO exposure demonstrated a β of −3.32 (95% CI: −4.89, −1.74, p < 0.0001), indicating lower HDL levels. This association persisted in model 3, with a β of −3.57 (95% CI: −5.18, −1.96, p < 0.0001). Similarly, the continuous log-transformed HbEO variable was inversely associated with HDL (β = −3.14, 95% CI: −4.41, −1.87, p < 0.0001), showing a consistent negative relationship across models.

**TABLE 3:** Multiple linear regression associations of HbEO with lipid profiles in adults.

Lipid profiles	Model	Continuous log10-transformed EO	Quartile1	Quartile2	Quartile3	Quartile4	p for trend
			β	β(95%CI)	β(95%CI)	β(95%CI)	
HDL (n = 3444)	Model1	−2.26 (−3.46, −1.07)***	0.00(Ref.)	−1.96 (−3.54, −0.39)*	−2.16 (−3.73, −0.58)**	−3.32 (−4.89, −1.74)***	<0.0001
	Model2	−3.33 (−4.43, −2.24)***	0.00(Ref.)	−1.31 (−2.70, 0.08)	−2.37 (−3.78, −0.96)**	−3.91 (−5.34, −2.48)***	
	Model3	−3.14 (−4.41, −1.87)***	0.00(Ref.)	−0.82 (−2.19, 0.55)	−1.53 (−2.92, −0.13)*	−3.57 (−5.18, −1.96)***	
TC (n = 3444)	Model1	−4.28 (−7.21, −1.36)*	0.00(Ref.)	−1.61 (−5.46, 2.24)	−1.05 (−4.90, 2.80)	−4.41 (−8.27, −0.56)*	0.5704
	Model2	−1.03 (−4.03, 1.97)	0.00(Ref.)	−1.86 (−5.68, 1.95)	−0.78 (−4.65, 3.08)	−0.58 (−4.51, 3.34)	
	Model3	−1.02 (−4.55, 2.51)	0.00(Ref.)	−1.29 (−5.09, 2.50)	0.51 (−3.37, 4.38)	−0.19 (−4.66, 4.28)	
TG (n = 1622)	Model1	−5.57 (−17.18, 6.04)	0.00(Ref.)	−5.91 (−21.32, 9.50)	−0.20 (−15.66, 15.27)	−7.72 (−23.20, 7.77)	0.4424
	Model2	5.59 (−6.27, 17.46)	0.00(Ref.)	−6.03 (−21.06, 9.00)	4.80 (−10.53, 20.14)	4.68 (−11.06, 20.43)	
	Model3	5.46 (−8.48, 19.40)	0.00(Ref.)	−7.45 (−22.58, 7.67)	3.20 (−12.34, 18.74)	3.90 (−13.91, 21.71)	
LDL (n = 1607)	Model1	−2.97 (−6.72, 0.78)	0.00(Ref.)	−0.65 (−5.65, 4.34)	−0.20 (−5.20, 4.81)	−2.07 (−7.08, 2.94)	0.9431
	Model2	−0.61 (−4.53, 3.30)	0.00(Ref.)	−0.46 (−5.44, 4.52)	0.58 (−4.49, 5.65)	0.90 (−4.31, 6.10)	
	Model3	0.17 (−4.39, 4.73)	0.00(Ref.)	0.41 (−4.56, 5.38)	2.06 (−3.04, 7.15)	2.40 (−3.43, 8.23)	
AIP(n = 1622)	Model1	0.02 (−0.02, 0.05)	0.00(Ref.)	0.02 (−0.03, 0.06)	0.02 (−0.03, 0.07)	0.02 (−0.03, 0.07)	0.0020
	Model2	0.08 (0.04, 0.11)***	0.00(Ref.)	0.02 (−0.03, 0.06)	0.05 (0.01, 0.10)*	0.09 (0.04, 0.13)***	
	Model3	0.06 (0.02, 0.10)**	0.00(Ref.)	0.01 (−0.04, 0.05)	0.04 (−0.00, 0.08)	0.07 (0.02, 0.12)*	

Model 1 was not adjusted.

Model 2 was adjusted for age, gender, race, and BMI.

Model 3 was adjusted as Model 2 plus education level, PIR, vigorous work activity, smoking status, alcohol consumption, diabetes, and hypertension.

The associations between HbEO and TC, TG, and LDL were generally non-significant after adjustments, with no distinct patterns emerging. Notably, AIP, a novel lipid marker, showed a statistically significant association with EO in the fully adjusted model, despite the limited effect size. The β coefficient for the log-transformed EO and AIP was 0.06 (95% CI: 0.02, 0.10, p = 0.002). While this effect reached statistical significance, its overall impact remained moderate.

### HDL mediated the association between HbEO and multiple kidney parameters

**FIGURE 3:**
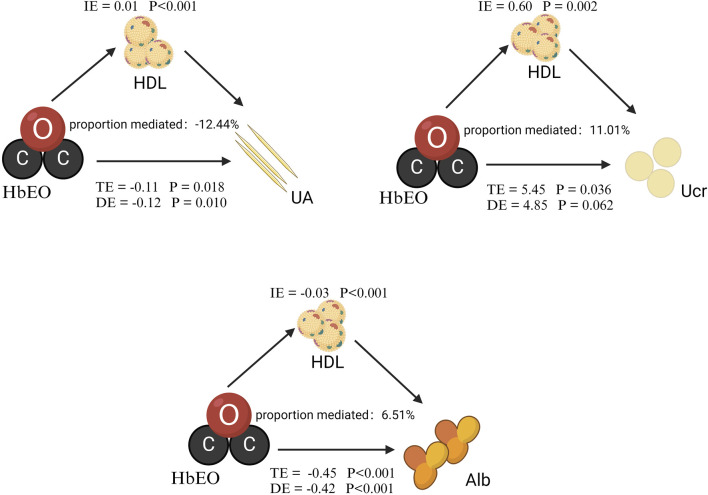
Mediation analysis of HDL on the interaction between HbEO and kidney parameters. The models were adjusted for gender, age, race, PIR, education level, alcohol consumption, smoking status, BMI, vigorous work activity, diabetes, and hypertension. Note: I.E., Indirect effect; TE, Total effect; DE, Direct effect.

We performed a mediation analysis to assess the influence of HDL on the relationships between HbEO levels and kidney parameters. As shown in Figure 3 and Table 4, HDL significantly mediated these relationships, accounting for 6.51% of the variation in Alb, −12.44% in UA, and 11.01% in Ucr. Although partial mediation was observed, the negative mediation effect observed for UA indicates a complex relationship that may require further exploration. Overall, these findings highlight a modest mediating effect of HDL in the association between EO levels and kidney parameters. While this result is correlational, causal relationships warrant further investigation.

**TABLE 4:** The mediation effects of HDL on the association of log10-transformed HbEO with kidney parameters in adults.

Kidney parameters	Mediator	Indirect effects	Direct effects	Total effects	Mediated proportion (%)	p value
		β (95%CI)	β (95%CI)	β (95%CI)		
Alb	HDL	−0.03 (−0.05, −0.01)***	−0.42 (−0.65, −0.20)***	−0.45 (−0.67, −0.24)***	6.51%	<0.001
UA		0.01 (0.01, 0.02)***	−0.12 (−0.20, −0.03)*	−0.11 (−0.19, −0.01)*	−12.44%	0.02
Ucr		0.60 (0.19, 1.18)**	4.85 (−0.19, 10.06)	5.45 (0.51, 10.61)*	11.01%	0.04

Model was adjusted for age, gender, education level, race, PIR, BMI, vigorous work activity, smoking status, alcohol consumption, diabetes, and hypertension.

## Discussion

Previous research indicates that ethylene oxide exposure exerts multisystemic effects, with documented impacts on respiratory function, hematological parameters, endocrine regulation, and reproductive health (Agency for Toxic Substances and Disease Registry ATSDR Toxicological Profiles, 2022; Lewis et al., 1986). Additionally, EO has been linked to tumor development. A two-year inhalation study demonstrated significant increases in benign and malignant tumors in B6C3F1 mice exposed to 50 ppm and 100 ppm EO, confirming its carcinogenic potential (NTP Toxicology and Carcinogenesis Studies, 1987). In the renal system, researchers reported kidney abnormalities induced by EO exposure, including enlargement, mild congestion, and cloudy swelling in the convoluted tubules of both rats and guinea pigs (Hollingsworth et al., 1956). Furthermore, studies indicate that repeated EO exposure increases lipid peroxidation and alters glutathione metabolism in the liver, as evidenced by elevated malondialdehyde (Katoh et al., 1989). While most EO data derived from animal studies, recent work focuses on human exposure-health linkages. For example, Li et al. (2024) reported significant correlations between elevated HbEO and liver damage markers, while Huang et al. (2023) observed a J-shaped relationship between EO exposure and the risk of developing chronic obstructive pulmonary disease. Moreover, cardiovascular conditions like angina, heart attacks, and overall CVD have been associated with EO, and inflammation mediates these effects (Zhou W. et al., 2024; Zeng et al., 2021; Cheang et al., 2022). Nevertheless, the effects of EO exposure on renal function and lipid metabolism in general human populations have not been comprehensively demonstrated.

In this study, HbEO levels were associated with various kidney parameters. Higher HbEO levels correlated with lower Alb. BUN showed negative associations with log10-transformed HbEO but positive associations in lower quartiles. Both the highest quartile of HbEO and continuous log10-transformed values demonstrated an inverse relationship with uric acid. The UA/Scr ratio displayed a similar pattern. RCS analysis indicated nonlinear relationships between HbEO and BUN, UA, UA/Scr, and Alb. Higher HbEO exposure was also associated with lower HDL levels. Mediation analysis found that HDL contributed to 6.51% of the Alb reduction, −12.44% of the UA decline, and 11.01% of the Ucr elevation.

The inverse association between HbEO and Alb observed in our study aligns with prior reports of EO-induced renal impairment in CKD populations (Wu et al., 2024). This consistency is further reinforced by existing evidence demonstrating EO-related albuminuria risk elevation (Zhou W. et al., 2024), providing a potential explanatory framework for the Alb findings in this study. The observed lipid disturbances gain biological plausibility from established literature documenting EO’s disruptive effects on metabolic pathways, particularly lipid metabolism (Sun et al., 2025; Cheang et al., 2022; Zhao et al., 2024). Published mechanistic studies support HDL’s renoprotective role during systemic toxic exposures (Sun et al., 2015; Wang et al., 2024), suggesting its potential mediation in EO-related renal effects. Interestingly, we observed inverse associations between HbEO levels and certain conventional renal markers (BUN and UA)—parameters that typically increase with renal dysfunction (Heuchel et al., 2023; Johnson et al., 2013; Kanbay et al., 2010). This contrasts with the well-documented association between EO exposure and elevated CKD risk (Wu et al., 2024).

Notably, while prior CKD research focused on clinically confirmed cases with manifest renal dysfunction, this population-based study reveals early-stage adaptive responses to EO exposure. A plausible explanation for the reduced UA and BUN levels is that, akin to certain environmental toxins (Orr and Bridges, 2017; Petejova et al., 2019), EO might induce compensatory mechanisms (e.g., enhanced renal excretion) during early or high-exposure phases, thereby leading to reduced UA and BUN levels. It has been suggested that EO can react with amino acid residues, such as cysteine and methionine, potentially leading to protein degradation through adduct formation with Alb (Funatsu et al., 2019). Given the observed association between HbEO levels and reduced Alb in our study, future research could explore whether the formation of such adducts plays a role in altering Alb levels.

Moreover, EO exposure has been linked to DNA damage in various tissues, including the kidneys (Zhang et al., 1997). Specifically, repeated EO exposure in animal studies has resulted in the accumulation of DNA adducts like HEtVal and 7-HEG in multiple organs (Walker et al., 1993; Rusyn et al., 2005). Future studies could examine if these adduct-mediated genomic alterations mechanistically underlie the renal biomarker changes in EO-exposed humans. EO has also been reported to form adducts with hemoglobin, raising questions about its long-term effects on the hematological system (Walker et al., 1993).

Inflammatory pathways may represent another mechanism linking EO exposure to renal dysfunction. Multiple studies have highlighted the role of inflammation in EO-related kidney damage and proteinuria (Wu et al., 2024; Zhou W. et al., 2024). However, its mediating role between EO exposure and kidney function markers has yet to be fully explored. Inflammatory processes are known to affect kidney markers (Chang et al., 2022; Graterol Torres et al., 2022). For example, UA has been implicated in both acute kidney injury and chronic kidney disease, with inflammation driving increased UA levels (Jung et al., 2020; Kang et al., 2005). Nevertheless, the potential involvement of inflammatory mediators in the observed association between EO exposure and alterations in early renal biomarkers necessitates additional mechanistic studies.

Alterations in lipid parameters observed in this study may be related to oxidative stress and inflammation associated with EO exposure. Numerous animal studies have shown that EO exposure leads to increased glutathione consumption and enhanced lipid peroxidation in the liver, both of which can aggravate oxidative stress (Katoh et al., 1989; Katoh et al., 1988). These mechanisms plausibly contribute to the HDL reduction observed in our study. Emerging evidence suggests an association between EO exposure and lipid alterations mediated through inflammatory pathways (Zhu et al., 2022). Changes in lipid levels, particularly increases in TG and decreases in HDL, are often closely linked to inflammation (Curley et al., 2021). Inflammatory cascade activation elevates TG and suppresses HDL, thereby disrupting cholesterol transport (Esteve et al., 2005). Further research is needed to elucidate the precise mechanisms by which EO may contribute to reductions in HDL. Beyond its role in lipid metabolism, HDL has also been proposed to exert protective effects on renal parenchymal cells through its antioxidant and anti-inflammatory functions (Zhong et al., 2019). However, the negative indirect effect of HDL on Alb levels in our mediation analysis indicates that its role may involve mechanisms beyond renal protection, warranting further exploration. Although the direct effect of EO on Ucr demonstrated marginal statistical significance, mediation analysis was conducted given its unique capacity to reveal mechanistic pathways that conventional direct association analyses might overlook.

In conclusion, this study examines the associations between EO exposure and various kidney and lipid parameters, highlighting the regulatory role of HDL. Our investigation into the nonlinear relationships between EO and kidney parameters, including UA, BUN, UA/Scr, and Alb, offers evidence contributing to the understanding of the complex biological processes involved. The findings underscore the importance of lipid markers, particularly HDL, in mediating EO’s effects on kidney health, providing a foundation for further exploration into EO’s broader systemic impacts. Although the observed effect sizes were moderate, they may still be of clinical concern given the widespread exposure to EO; further studies are needed to clarify the exposure-risk threshold. The analysis utilized a large population dataset, with careful adjustments for multiple potential confounders to enhance the robustness of the models. However, several limitations warrant consideration. The cross-sectional design of this study precludes causal inference, limiting the findings to demonstrated associations rather than definitive relationships. Additionally, the specific demographic and environmental characteristics of the NHANES dataset may restrict the generalizability of results to broader populations. Furthermore, the use of complete-case analysis raises potential selection bias if excluded participants systematically differed from those included in the analysis. Finally, residual confounding remains possible due to unmeasured or imperfectly controlled factors, such as genetic predisposition, socioeconomic status, or environmental co-exposures, that could influence both exposure and outcomes. Collectively, these limitations underscore the need for longitudinal studies to validate the findings and elucidate the underlying biological mechanisms.

## Conclusion

This study demonstrates significant associations between EO exposure and various markers of kidney function and lipid metabolism. The results indicate EO may affect renal physiology and lipid regulation through mechanisms more complex than direct toxicity alone. Further research is needed to clarify the underlying biological pathways and dose-response relationships. These results underscore the importance of public health vigilance regarding EO exposure and its potential multi-system effects.

## Data Availability

The original contributions presented in the study are included in the article/supplementary material, further inquiries can be directed to the corresponding authors.

## Abbreviations

AIP, Atherogenic index of plasma; Alb, Serum albumin; BMI, Body mass index; BUN, Blood urea nitrogen; CVD, Cardiovascular disease; EO, Ethylene oxide; HbEO, Hemoglobin adducts of ethylene oxide; HDL, High-density lipoprotein; LDL, Low-density lipoprotein; NHANES, National Health and Nutrition Examination Survey; PIR, Poverty income ratio; RCS, Restricted cubic spline; Scr, Serum creatinine; TC, Total cholesterol; TG, Triglycerides; UA, Uric acid; Ualb, Urinary albumin; Ucr, Urinary creatinine.
