# Correction: Investigating ethylene oxide exposure and its associations with kidney indicators and lipid profiles: the mediating effect of HDL in NHANES 2013–2020

**DOI:** 10.3389/fphys.2025.1633971

**Published:** 2025-06-12

**Authors:** Yiheng Luo, Duo Xu, Weiyong Zhang, Kai Wang, Mingqin Kuang, Yueyang Liu

**Affiliations:** ^1^ The First School of Clinical Medicine, Southern Medical University, Guangzhou, China; ^2^ School of Public Health, Southern Medical University, Guangzhou, China; ^3^ Department of Internal Medicine, Shenzhen TCM Anorectal Hospital (Futian), Shenzhen, China; ^4^ Department of Gynecology and Oncology, Ganzhou Cancer Hospital, Ganzhou, China; ^5^ Department of Gynecology, Guangdong Provincial People’s Hospital (Guangdong Academy of Medical Sciences), Southern Medical University, Guangzhou, China

**Keywords:** ethylene oxide, kidney parameters, lipid profiles, epidemiology, national health and nutrition examination survey

In the published article, there was an error in **Affiliations 3 and 4**. Instead of “(3Department of Gynecology and Oncology, Ganzhou Cancer Hospital, Ganzhou, China), (4Department of Internal Medicine, Shenzhen TCM Anorectal Hospital (Futian), Shenzhen, China)”, it should be “(3Department of Internal Medicine, Shenzhen TCM Anorectal Hospital (Futian), Shenzhen, China), (4Department of Gynecology and Oncology, Ganzhou Cancer Hospital, Ganzhou, China”).

The authors apologize for this error and state that this does not change the scientific conclusions of the article in any way. The original article has been updated.

